# Letter to the Editor: Surveillance of *mcr-1* and *mcr-2* genes in Carbapenem-resistant *Klebsiella pneumoniae* strains from an Italian Hospital

**DOI:** 10.2807/1560-7917.ES.2017.22.35.30604

**Published:** 2017-08-31

**Authors:** Carolina Venditti, Carla Nisii, Silvia D’Arezzo, Antonella Vulcano, Antonino Di Caro

**Affiliations:** 1 National Institute for Infectious Diseases (INMI) L. Spallanzani - IRCCS, Rome, Italy

**Keywords:** mcr-1, colistin, antibiotic-resistance

**To the editor:** In a recent editorial [1], Kluytmans reports on the rise of plasmid-mediated colistin resistance that has occurred worldwide since its first description in 2016 [2]. Antimicrobial resistance, especially in Gram-negative bacteria, has been recognised as a serious threat to human health, and colistin, as well as tigecycline, is often the only therapeutic option for infections caused by carbapenem-resistant *Enterobacteriaceae* (CRE). Resistance to colistin can occur in *Enterobacteriaceae* either through adaptive chromosomal mutations that alter the bacterial outer membrane, or through plasmid-mediated horizontal gene transfer. The recently recognised global distribution of plasmid-borne colistin resistance determinants *mcr-1,mcr-2*, *mcr-3* and *mcr-4* represent a concern for infection prevention and public health , especially in the context of high CRE prevalence in hospital wards [3-5].

Recent data indicate that colistin resistance occurs more frequently among CRE than carpapenem-susceptible strains, and a recent survey conducted in Italian hospitals showed a high rate (almost 40%) of colistin resistance among CRE, although *mcr*-1 was not detected by subsequent molecular tests [6]. In this context, we would like to share the preliminary results of an ongoing retrospective and prospective study aimed at detecting the presence of *mcr*-1 and *mcr*-2 in all our colistin-resistant isolates of carbapenem-resistant *Klebsiella pneumoniae*. We screened our collection of 369 strains isolated between 2013 to July 2017. Most were obtained from patients treated at our tertiary hospital in Rome, and some were referred from other hospitals of the Rome area. Reduced susceptibility to colistin was recorded for 127/369 (34%), with minimal inhibitory concentrations (MICs) ranging from 2 mg/L to greater than 16 mg/L. Given that some *mcr-1*–positive isolates may have a colistin MIC as low as 2 mg/L, we lowered our inclusion threshold to include this value. All isolates were screened by PCR for the presence of *mcr-1* and *mcr*-2 genes as previously described [2,3]. The percentage of colistin resistance in our carbapenem-resistant *K. pneumoniae* strains remained relatively constant over the years at around 30%, with the exception of 2016 when it dropped to 15%, and the first half of 2017 when we recorded 44% of resistant strains. None of the isolates studied so far were found to harbour the *mcr*-1 or *mcr*-2 genes, and our research now focuses on identifying the mechanisms that caused the observed colistin resistance.

We agree with Klytmans’ conclusion that improved surveillance efforts are warranted in humans, in animals and in the environment, especially given the recent discovery of *mcr-3*, *mcr-4, *and most likely further *mcr* resistance genes occurring in the future in Europe, and that *E. coli* strains carrying the *mcr-1* gene have been circulating in Italy at least since 2013 [7]. Our preliminary data confirm that plasmid-mediated colistin resistance in humans is still very low in the catchment area of our hospital in Italy. However, we need to urgently prevent what could become a nightmare scenario in the future, i.e. the loss of a drug that in many cases is considered a last resort.
